# Associations Between Physical Activity Frequency, Handgrip Strength, and Limitations in Activities of Daily Living in Middle-Aged and Older Adults with Widespread Pain: A Cross-Sectional Study Using Data from the SHARE Project

**DOI:** 10.3390/geriatrics10050125

**Published:** 2025-09-14

**Authors:** Ángel Denche-Zamorano, José Carmelo Adsuar, Sabina Barrios-Fernandez, Diana Salas-Gómez

**Affiliations:** 1 Promoting a Healthy Society Research Group (PHeSO), Faculty of Sport Sciences, University of Extremadura, 10003 Cáceres, Spain; 2 BioErgon Research Group, Faculty of Sport Sciences, University of Extremadura, 10003 Cáceres, Spain; 3 Social Impact and Innovation in Health (InHEALTH), Nursing and Occupational Therapy College, University of Extremadura, 10003 Cáceres, Spain

**Keywords:** exercise, independence, participation, pain, health system, sustainability

## Abstract

**Background:** The increase in life expectancy has led to a higher prevalence of chronic conditions, including widespread pain (WP), which often compromises functional independence and quality of life in older adults. WP is strongly associated with limitations in Basic and Instrumental Activities of Daily Living (BADLs and IADLs). While physical activity (PA) and muscle strength (handgrip) are known to enhance general health, their specific role in preserving functional capacity in individuals with WP remains underexplored. **Methods:** This cross-sectional study analyzed data from 1154 adults aged 50–80 reporting WP, drawn from the Ninth Wave of The Survey of Health, Ageing and Retirement in Europe (SHARE). Associations between moderate and vigorous PA (MPA and VPA), handgrip strength (HGS), and limitations in BADLs and IADLs were examined using multivariate logistic regression, adjusting for demographic and health-related variables. **Results:** Lower MPA was significantly associated with greater odds of all BADL and most IADL limitations, while VPA had no significant associations. Higher relative HGS was significantly associated with lower odds of limitations in BADLs and IADLs. **Conclusions:** Among older adults with WP, MPA and muscular strength appear to reduce the risk of functional limitations. These findings highlight the relevance of promoting sustainable strategies to support independence in aging populations.

## 1. Introduction

The world population is undergoing a demographic transition [1] with progressive global aging that is expected to continue in the coming decades [2]. Based on the World Health Organization (WHO) data from 2023 [3], about 14% of the world’s population was 60 years old or older, and this proportion is estimated to reach 22% by 2050 [4]. This fact is placing a greater burden on health and social care systems [5] as aging is associated with a higher prevalence of long-term illnesses (physical and psychological), multimorbidity, functional dependence, feelings of loneliness, cognitive impairment, greater risk of frailty, and increased mobility limitations, and it affects Activities of Daily Living (ADLs) [5,6,7,8,9].

Pain is closely related to aging, with a high prevalence of chronic pain in older people (27–58% in those over 65) [1]. Pain is a sensory and/or emotional experience that causes discomfort, either localized or generalized, related to physical or psychological stimuli, which can have multiple causes or origins [6]. Back, hip, and knee pain are the most prevalent types of localized pain. However, between 25 and 43% of this population may experience multi-site pain, i.e., pain in several locations at the same time [1]. Furthermore, aging and pain share a bidirectional connection: individuals with severe general pain tend to feel older than those without pain or with mild general pain, while individuals who feel older tend to report higher pain levels [7]. Aging also increases the impact of pain on health, functionality, and quality of life for those who suffer from it [8,9]. Both localized pain and multi-site pain are associated with limitations in Basic Activities of Daily Living (BADLs) [10,11], which refer to self-care activities [12] and include eating, dressing, personal hygiene, toileting, as well as functional mobility; and also limitations in Instrumental Activities of Daily Living (IADL) [11,13,14], comprising those that involve caring for others, home management, and community activity participation [12] and which can be subdivided into caring for pets, children, and others, managing communications, driving and moving within the community, managing financial resources, doing housework, preparing meals, shopping, carrying out safety procedures, and spiritual expression.

Widespread pain (WP) has a lower prevalence, around 10–15% of the population [15,16], but an even greater impact on quality of life and life expectancy [17,18]. This condition is treated when the pain cannot be localized in specific areas of the body and spreads diffusely over large areas of the body or throughout the whole body [15,19,20]. In the scientific literature, diverse research has reported that WP negatively affects the quality of life of those who suffer from it, having a strong relationship with increased risk of functional disability and limitations in ADLs [21,22].

In the older population, regular physical activity (PA), as recommended for older adults [23,24,25,26], is associated with multiple health benefits, including improved physical condition (cardiorespiratory fitness, strength, balance, and flexibility) and functionality (ability to perform ADLs), pain reduction, and prevention of age-related decline [25,26,27]. Within this group, handgrip strength (HGS) has been identified as a predictor of all-cause mortality, functional decline (higher risk of falls), and limitations in IADLs, including meal preparation, shopping, community mobility, and housekeeping [28,29,30,31]. Finally, the impact of pain on physical condition (e.g., cardiorespiratory fitness, muscle strength balance, and flexibility), functional capacity (i.e., the ability to perform ADLs and maintain functional independence), and quality of life appears to be modulated by both the PA level, with higher activity associated with lower impact, and the preservation of muscle strength.

For all the above, it is necessary to better understand the relationships between PA frequency (PAF), both moderate (MPA) and vigorous (VPA), and HGS strength in people with pain, especially those with WP. A better understanding of these relationships could help in the design of research and prevention and evaluation and intervention programs aimed at preserving ADL independence in people with WP. Thus, this study has the following main objectives:

Analyzing the relationships between PAF (MPA and VPA) and the limitations in ADLs (BADLs and IADLs) involves the following:

1. Comparing the prevalence of limitations in each of the BADLs and IADLs based on MPA and VPA.
2. Estimating the odds ratio (OR) of presenting limitations in each of the BADLs and IADLs based on PA: not performing MPA versus performing it very frequently, not performing VPA versus performing it very frequently, and HGS, all adjusted for age, sex, Body Mass Index (BMI), educational level, long-term illness, depressive symptoms, perception of loneliness, and cognitive functioning.

Based on these objectives, the study hypotheses are as follows:

1. Limitations in BADLs and IADLs will be associated with MPA and VPA frequency.
2. People with WP who perform MPA or VPA with some frequency will have lower ADL limitation prevalences than people who do not perform them or perform them rarely.
3. Performing MPA or VPA rarely or never will increase the likelihood of limitations in each of the ADLs, while greater relative HGS will be a protective factor against the onset of such limitations.

## 2. Materials and Methods

### 2.1. Study Design and Ethical Considerations

The study is a secondary cross-sectional study based on data from the Ninth Wave of The Survey of Health, Ageing and Retirement in Europe (SHARE). This project started in 2004 and has been carried out every two years, analyzing the processes associated with aging in adults in European countries [32,33,34]. During the first four waves, the project was evaluated and approved by the Ethics Committee of the University of Mannheim, while, from the fifth wave onwards, the ethical review was carried out by the Ethics Committee of the Max Planck Society.

### 2.2. Share Data Collection

The Ninth Wave of the SHARE used a probabilistic sampling method for sample selection, following the same methodology used in the Eighth Wave. Households in which at least one adult member spoke the official language of the country and did not reside outside the country during the data collection period were included. In addition to the longitudinal samples from previous waves and the national replacement groups already included in the Eighth Wave, new national replacement groups were incorporated that had previously been unable to participate due to the interruption of fieldwork caused by COVID-19. Eligibility criteria are described in detail in the SHARE Release Guide, which is publicly available on the official SHARE-ERIC website (Share Methodology). Data collection took place between October 2021 and September 2022. The interviews with participants were conducted in person, involving standardized interviews carried out by trained staff using computer-assisted interview systems (CAPI). These interviews were conducted both in private homes and in nursing homes. Participants who gave their consent completed a face-to-face main interview that addressed various topics, such as sociodemographic information, and physical, psychological, and cognitive health status, among others. The complete methodology of the study can be found on the official SHARE-ERIC website [35].

### 2.3. Sample Selection and Eligibility Criteria

The files and responses used in the analysis for this study are available upon request through The SHARE Data Platform (https://share-eric.eu/data/ accessed on 1 March 2020). Once access was granted, data were downloaded in a format compatible with the IBM SPSS Statistics v.27 (IBM Corp, Armonk, NY, USA) software. The initial sample corresponding to the people who responded to the Ninth Wave of the SHARE study survey consisted of 69,447 participants, including participants from European countries such as Germany, Austria, and Belgium, as well as other nations in Central, Northern, and Southern Europe, in addition to Israel.

**Figure 1:**
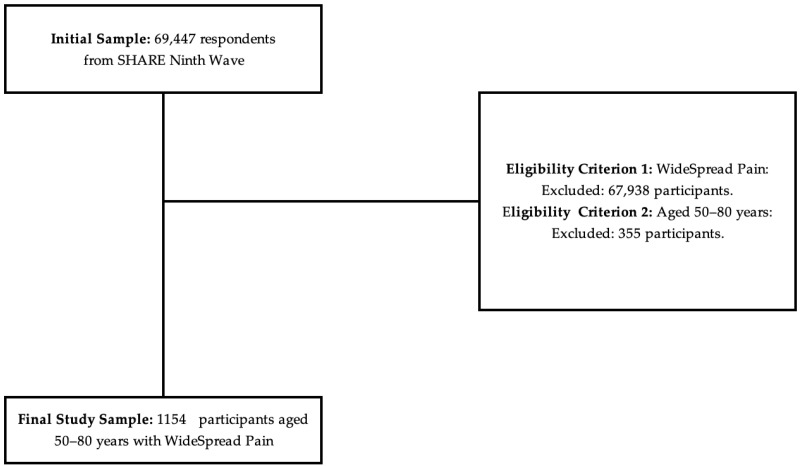
Sample selection flowchart.

The following eligibility criteria were established for this study: (1) answering ‘Yes’ to the question ‘Are you troubled with pain?’; (2) answering the question about pain with ‘All over Pain’; and (3) being between 50 and 80 years old. After applying these criteria, the sample was composed of 1154 participants between 50 and 80 years (Figure 1).

### 2.4. Variables

#### 2.4.1. Characterization Variables

Sociodemographic data included age (in years), sex (male/female), height (in centimeters), and body weight (in kilograms). BMI was calculated from each participant’s height and weight using the formula kg/m^2^, resulting in four categories: underweight (BMI < 18.5 kg/m^2^), normal weight (BMI ≥ 18.5 and < 25 kg/m^2^), overweight (BMI ≥ 25 and < 30 kg/m^2^), and obesity (BMI ≥ 30 kg/m^2^) [36].

Educational level was also considered, using the International Standard Classification of Education (ISCED-97), which provides the following options: no education, pre-school education, primary education, lower secondary education, upper secondary education, post-secondary non-tertiary education, first-cycle tertiary education, and people still studying [37].

Regarding chronic diseases, participants were given an explanation about what is meant by long-term illness, defining it as a health problem that persisted over time. Then, they were asked if they had any illness, condition, disability, or health problem of this type, the possible answers being ‘Yes’ or ‘No’.

Physical condition was assessed based on PAF and HGS. On the one hand, PA was categorized into two types based on energy expenditure: moderate (MPA) and vigorous (VPA). Participants were asked how often they engaged in activities that required moderate physical effort (MPA), such as gardening, washing the car, or going for a walk, the response options including more than once a week, once a week, one to three times a month, or almost never or never; and VPA, such as playing sports, doing heavy housework, or performing jobs that required considerable physical effort, with the same response as in the previous section. Participants could meet the criteria for both MPA and VPA, only one of them, or neither; these categories were therefore not mutually exclusive.

On the other hand, HGS was evaluated using a portable dynamometer (Smedley model, S Dynamometer, TTM, Tokyo, Japan), with a capacity of up to 100 kg. The procedure included asking the participants to exert as much force as possible when gripping the dynamometer. Two measurements were taken for each hand and were considered valid if the difference between the two did not exceed 20 kg in the same hand, following criteria established in previous studies. Subsequently, the highest grip value was recorded. Finally, this value was adjusted concerning body weight expressed as a ratio (kg/weight in kg) [38,39].

Regarding mental health, data on depressive symptoms and perceptions of loneliness were collected. Self-reported depressive symptoms were assessed using The EURO-D Measure of Depressive Symptoms in the Aging Population Scale [40]. This tool consists of 12 items that address different emotional manifestations, such as sadness, pessimistic view of the future, suicidal thoughts, feelings of guilt, sleep disturbances, loss of interest, irritability, changes in appetite, fatigue, difficulty concentrating, inability to enjoy oneself, and tendency to cry. Each item identifies the presence or absence of every symptom (1/0), allowing for a total score between 0 and 12, with higher values indicating a greater number of depressive symptoms. For the analysis, the scores were dichotomized: 0 to 3 points were considered an absence of depressive symptoms, and 4 or more points were interpreted as the presence of such symptoms. The perception of loneliness was measured using the Three-Item Loneliness Scale [41], designed to assess the degree of perceived social connection. This scale has shown high internal consistency in previous research (α = 0.82) [42]. Participants were asked to indicate how often they experienced feeling unaccompanied, feeling excluded, or feeling isolated from others. Responses were recorded on a 3-point Likert scale, ranging from 1 (almost never) to 3 (frequently). The sum of the responses resulted in a total score ranging from 3 to 9, with higher values indicating a greater level of perceived loneliness.

Cognitive functioning [43] was assessed based on the sum of results achieved in several cognitive tests, up to a maximum of 29 points. These tests included temporal orientation, by identifying the date, day of the week, month, and year (1 point for each correct answer); memory, using immediate and delayed recall of a list of 10 words (1 point for each modality); and executive function, by performing five consecutive mathematical calculations (successive subtractions, 1 point for each correct answer).

#### 2.4.2. Dependent Variables

Dependent variables included ADLs, both BADLs and IADLs. In the SHARE Project, independence in performing BADLs was assessed using a Katz Index modification [44] to which participants responded in a dichotomous format (yes/no) whether they had difficulty performing them. Activities included dressing (including socks and shoes); functional mobility (inside the home); transferring (getting out of bed or lying down); bathing; feeding/eating (e.g., cutting food); and toileting and toilet hygiene (including sitting down and getting up from the toilet). IADLs were measured using a modification of the Lawton and Brody Scale [45] for the SHARE Project, again with dichotomous responses (yes/no) regarding the difficulty in performing them and including meal preparation (hot meal); shopping; ability to use the phone; responsibility for medication; housekeeping (performing household or gardening tasks); financial management; community mobility (leaving home and using public transport unaccompanied); and doing laundry.

### 2.5. Statistical Analysis

Analyses were performed using IBM SPSS Statistics version 27 software, establishing a significance level of *p* < 0.05. The Kolmogorov–Smirnov test was used to test the normality of the continuous variables. Descriptive statistics were calculated for all study variables, with continuous variables expressed as medians (mdns) and interquartile ranges (IQRs) and categorical variables expressed as absolute frequencies and percentages.

To analyze the relationship between sex and the continuous sample characterization variables, the Mann–Whitney U test (UM) was carried out, and, for categorical variables, the Chi-square test (*χ*^2^) was performed. To analyze the relationship between each of the ADLs (BADLs and IADLs) and PAF (MPA and VAF), the Chi-square test was also used. In case of significant differences, a post hoc analysis of independent proportion differences was applied with the Bonferroni adjustment. The strength of the association was evaluated using Cramer’s V coefficient (V).

Multiple binary regression models were constructed to assess whether the difficulty presented in each BADL and IADL was associated with the physical condition variables (MPA and VPA, and HGS ratio). For this purpose, every ADL was introduced into each model as a dependent variable and physical condition variables as predictors. Two models were created for each ADL, one with MPA and the other with VPA. Odds ratios (ORs) with 95% confidence intervals were calculated for each predictor variable (PAF and HGS ratio) and adjusted for age, sex, BMI, educational level, long-term illness, depressive symptoms, perception of loneliness, and cognitive functioning.

### 2.6. GenAI Declaration

Generative Artificial Intelligence (ChatGPT, GPT-4 model, OpenAI; and DeepL Translator, DeepL SE) has been used in this paper to refine the language of the manuscript and improve the clarity and communication of complex ideas.

## 3. Results

### 3.1. Descriptive Results

The continuous variable data did not follow a normal distribution (*p* < 0.05). A total of 1154 individuals composed the sample, of whom 71% were women and 29% were men. The median age was 68 years, and the IQR was 12 in the total sample. The men were significantly older than the women (mdn 70 vs. 68 years; UM = 124,866; *p* = 0.016).

Regarding perception of loneliness, no significant differences were observed between the sexes (mdn in both groups: 4; IQR = 3; UM = 103,751; *p* = 0.139). Significant differences were found in relative HGS, which was higher in men (mdn = 0.44; IQR = 0.18) than in women (mdn = 0.33; IQR = 0.15; UM = 41,414; *p* < 0.001). Cognitive functioning was higher in women (mdn = 17; IQR = 7) than in men (mdn = 16; IQR = 7; UM = 91,449.5; *p* < 0.001). Regarding symptoms related to depression, significant differences were observed between the sexes (*χ*^2^ = 6.60; df = 1; *p* = 0.012). Indeed, 63% of women and 54% of men presented depressive symptoms. No significant differences were found in the presence of long-term chronic diseases (*χ*^2^ = 0.63; df = 1; *p* = 0.426), which affected 87% of the total sample. Regarding MPA, significant differences were found between the sexes (*χ*^2^ = 10.65; df = 3; *p* = 0.014), being more frequent in women: 50% performed MPA more than once a week compared to 47% of men. No significant differences were found in the VPA frequency (*χ*^2^ = 3.64; df = 3; *p* = 0.303), with most of the sample reporting ‘rarely or never’. The full descriptive analysis can be found in Table 1.

### 3.2. Physical Activity Frequency and Basic Activities of Daily Living

**Figure 2:**
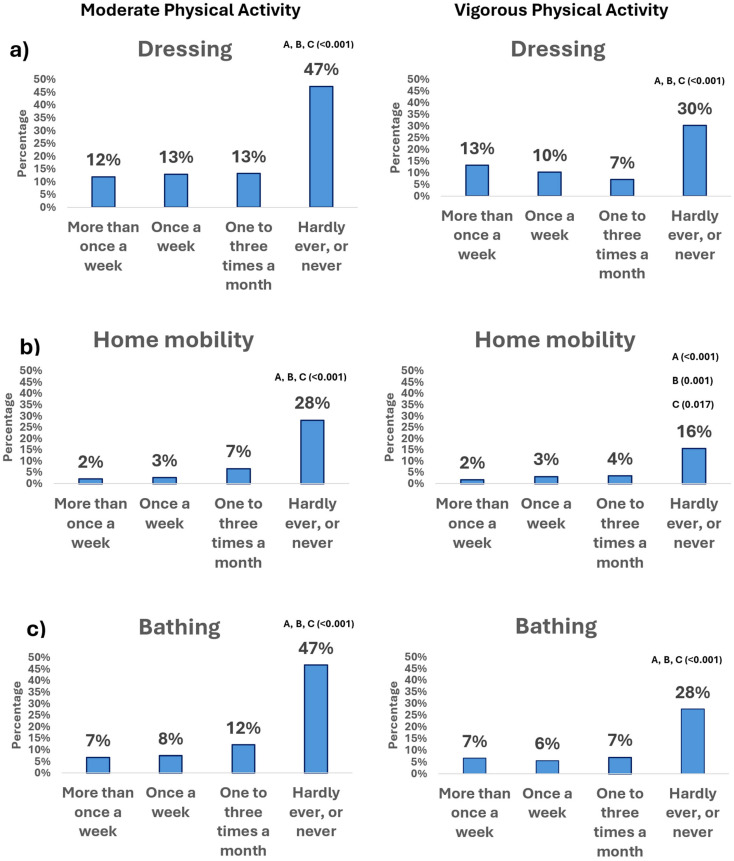

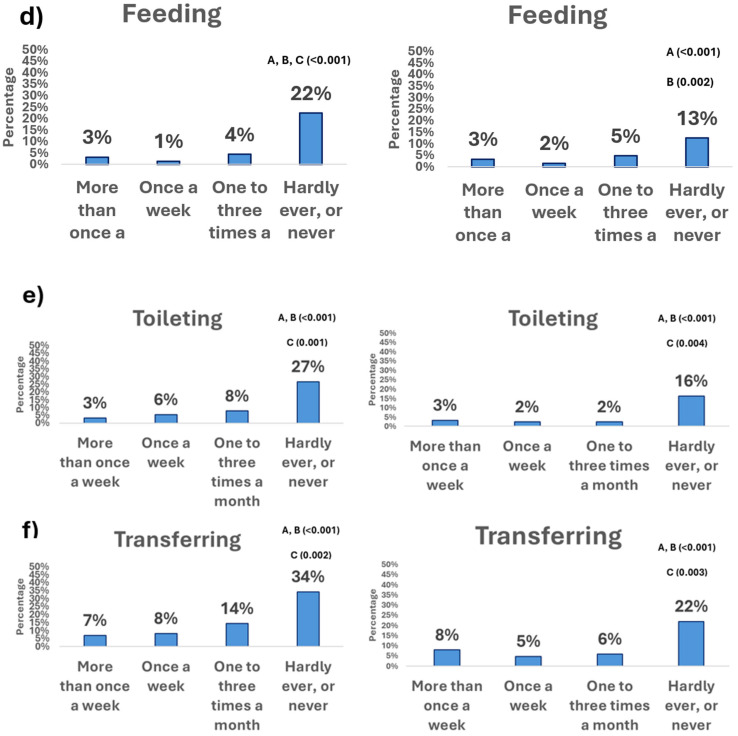
BADL limitations according to PAF (MPA—left, and VPA—right): Each column shows the percentage of participants within each PAF category (out of 100% in that category) who reported limitations (“Yes”). Letters indicate significant differences in the proportion of reported limitations among people with widespread pain according to PAF: **A** more than once a week; **B** once a week; **C** 1–3 times a month; **D** hardly ever or never.

Figure 2 shows the frequency with which the participants presented difficulties or limitations in the following BADLs: (a) dressing; (b) home mobility; (c) bathing; (d) feeding; (e) toileting; (f) transferring, according to PAF, divided into MPA and VPA.

#### 3.2.1. Moderate Physical Activity

A significant association was detected between MPA and the presence of limitations in BADLs (*p* < 0.05). As shown in Figure 2, in most BADLs, differences were found in the proportions of performance limitations between those who performed MPA ‘rarely or never’ and those who performed it more than once a week, once a week, or one to three times a month (*p* < 0.05). For example, those who ‘rarely or never’ performed MPA reported BADL difficulties with prevalences ranging from 22% (feeding) to 47% (bathing). In contrast, in these BADLs, only between 3 and 12% of those who performed MPA more than once a week experienced difficulty (*p* < 0.05). The findings are consistent across the other ADLs evaluated (Figure 2).

#### 3.2.2. Vigorous Physical Activity

The data analysis also revealed a significant association between VPA frequency and the presence of difficulties in various BADLs. The findings show that those participants who reported performing VPA less frequently had a higher prevalence of difficulties in performing BADLs. Concretely, between 13% (feeding) and 30% (dressing) of those who ‘rarely or never’ performed VPA reported difficulties in BADLs (*p* < 0.05). In contrast, in these BADLs, only between 3 and 13% of those who performed VPA more than once a week had limitations (*p* < 0.05). The results remained consistent in the other BADLs (Figure 2).

### 3.3. Physical Activity Frequency and Instrumental Activities of Daily Living

Statistically significant associations were observed between PAF and limitation prevalence in IADLs, regarding both MPA and VPA (*p* < 0.001 in all cases). In both modalities, lower PAF was associated with higher limitation prevalence in all the analyzed AIVDs. Specific data for each IADL are shown in Table 2.

### 3.4. Multivariate Logistic Binary Adjusted Regression

After adjusting for probability risks by age, sex, BMI, educational level, long-term illness, depressive symptoms, perception of loneliness, and cognitive functioning, those participants who never performed MPA had a higher risk of reporting BADL limitations compared to those who performed it very frequently (more than once a week). Specifically, they were more likely to have difficulties in dressing (OR = 2.55; 95% CI: 1.44–4.50; *p* = 0.001), home mobility (OR = 9.08; 95% CI: 2.99–27.55; *p* < 0.001), bathing (OR = 4.52; 95% CI: 2.34–8.73; *p* < 0.001), feeding (OR = 5.00; 95% CI: 1.61–15.49; *p* = 0.005), transferring (getting out of bed) (OR = 2.28; 95% CI: 1.13–4.62; *p* = 0.022), and toileting (OR = 2.69; 95% CI: 1.04–6.97; *p* = 0.042). In contrast, VPA frequency did not show statistically significant associations with BADLs (*p* > 0.05). In addition, greater relative HGS was associated with protection in all the BADLs, with ORs tending to zero (Table 3).

A multivariate logistic regression showed that performing MPA ‘rarely or never’ was significantly associated with a higher risk of limitations in several IADLs, including preparing hot meals (OR = 4.05; 95% CI: 1.57–10.48, *p* = 0.004), shopping (OR = 3.88; 95% CI: 2.03–7.40, *p* < 0.001), ability using the telephone (OR = 12.10; 95% CI: 1.76–83.29, *p* = 0.011), housekeeping (OR = 2.97; 95% CI: 1.82–4.86, *p* <0.001), financial management (OR = 3.14; 95% CI: 1.21–8.18, *p* = 0.019), responsibility for medication (OR = 9.20; 95% CI: 2.20–38.47, *p* = 0.002), community mobility (OR = 4.97; 95% CI: 2.62–9.42, *p* < 0.001), and doing laundry (OR = 7.53; 95% CI: 2.97–19.06, *p* < 0.001). In line with the findings regarding the BADLs, VPA was not significantly associated with any IADLs. Meanwhile, greater relative HGS was protectively associated with almost all the activities assessed, with ORs ranging from 0.00 to 0.02; *p* < 0.05 (Table 4).

## 4. Discussion

### 4.1. Main Findings

This study offers evidence on the relationship between PAF (MFA and VFA), muscular strength (HGS), and ADL performance (BADL and IADLs) in older adults with WP, emphasizing their relevance in preventing dependency and promoting sustainable aging. Among the main findings, it was found that infrequent engagement in MPA was associated with a higher risk of limitations in BADLs (dressing, bathing, and feeding) and IADLs (preparing meals, shopping, and responsibility for medication). In contrast, VPA showed no significant associations, reinforcing the notion that MPA may indicate a more suitable option for this population given its greater accessibility, adaptability, and feasibility [46,47]. Furthermore, higher relative HGS emerged as a protective factor, highlighting the importance of preserving muscular capacity to support independence in daily functioning.

In this study, older adults with WP from 50 to 80 years of age showed sociodemographic and clinical profiles in line with previous waves of the SHARE Project and previous research. In the analyzed sample, more than half of the participants were overweight or obese, in line with studies linking a higher BMI with a greater likelihood of persistent pain and loss of functionality in ADLs [10,15,48,49,50]. This study sample also showed a high prevalence of moderate to severe depressive symptoms and a moderate perception of loneliness, which was consistent with other studies that highlight the role of these factors in functional impairment in older adults with pain [25,51,52]. Pain and cognitive impairment are also related to pain [53,54,55,56,57], as well as chronic diseases [58,59,60]. All this reinforces the need to address pain, and, therefore, WP, from a comprehensive perspective that considers the physical, cognitive, emotional, and social determinants that condition ADL performance, social participation, and quality of life [61,62].

A higher MPA frequency was associated with a lower probability of limitations in both ADL subgroups: for BADLS, home mobility (OR = 9.08), feeding (OR = 5.00), bathing (OR = 4.52), toileting (OR = 2.69), dressing (OR = 2.55), and transferring (OR = 2.25); and in IADLs: shopping (OR = 3.88) and preparing meals (OR = 5.05); while in IADLs higher odds were observed in the ability to use the phone (OR = 2.10), responsibility for medication (OR = 9.20, doing laundry (OR = 7.53), community mobility (OR = 4.97), preparing meals (OR = 4.05), shopping (OR = 3.88), financial management (OR = 3.14), and housekeeping (2.97). These results are consistent with previous research highlighting the positive relationship between regular MPA and better functional performance in older adults with pain. Singh (2024) found that low PA levels were associated with greater ADL difficulties in older adults with musculoskeletal and multisite pain [1]. Therefore, physical inactivity, common in this population group [61], is associated with increased frailty and functional decline, contributing to a negative pain cycle characterized by reduced movement and progressive loss of ADL independence [10,63,64]. However, no significant associations were found between VPA and ADL limitations, either in BADL or IADL. This may be related to several factors. Older adults with pain, especially WP, have a lower tolerance to intense physical exertion due to persistent fatigue, functional impairment, and fear of exacerbating pain [10,58]. Additionally, multiple perceived barriers to vigorous exercise have been reported, including low self-efficacy, fear of falling, and beliefs that high-intensity activity may worsen their condition [65,66,67]. Older adults with chronic or WP can exhibit significant difficulties in both BADLs and IADLs.

The ADLs with the highest likelihood of showing limitations in physically inactive older adults with WP were using the telephone (OR = 12.10), responsibility for medication (OR = 9.20), home mobility (OR = 9.08), and doing laundry (OR = 7.53). Therefore, when compared, IADLs exhibited higher ORs than BADLs overall, suggesting that IADLs are more sensitive to the adverse effects of physical inactivity. IADLs are structurally more complex than BADLs, involving higher cognitive, sensorimotor, social, contextual, and cultural demands, which may help to explain the greater vulnerability of IADLs to the effects of physical inactivity [12]. These findings are in line with previous research. In a longitudinal study in India, 33.1% of the participants with pain reported difficulty with BADLs, while 57.1% reported difficulties with IADLs [68]. Another study with older adults from Australia with osteoporosis, osteoarthritis, and chronic back pain found that the most difficult ADL was ‘heavy housework’ (49.8%) [69].

Our results also show that higher HGS is associated with a lower probability of BADL and IADL limitations. The adjusted ORs show a consistent protective pattern in all the assessed activities (BADLs: OR = 0.03 in dressing, OR = 0.01 in bathing, OR = 0.00 in feeding, OR = 0.00 in toileting, and OR = 0.03 in transfers; IADLs: OR = 0.02 in meal preparation, OR = 0.03 in responsibility for medication, and OR = 0.02 in shopping). These findings underscore the importance of preserving muscular strength in older adults, particularly those with widespread pain, as a key factor in maintaining functional independence and reducing the risk of ADL dependency. This evidence aligns with previous studies that identify HGS as a reliable indicator of functionality in older adults. A study using data from the SHARE and the English Longitudinal Study of Ageing (ELSA) found that low cumulative HGS levels were associated with lower daily functioning (β: 0.267; 95% CI: 0.161, 0.374 for the ELSA and 0.153; 0.079, 0.227 for the SHARE) and a more rapid decline in daily functioning over time (0.105; 0.081, 0.129 for the ELSA, and 0.217; 0.195, 0.238 for the SHARE) [70]. Another study of 2038 community-dwelling older adults (aged 60–106) found that each additional kilogram of HSG was associated with a 1.2% reduction in the likelihood of experiencing pain, after adjusting for age, sex, BMI, and comorbidities (OR = 0.988; 95% CI: 0.980–0.995; *p* = 0.002) [71]. A 10-year longitudinal study based on the Health and Retirement Study (HRS) found that weakness in HGS was significantly associated with an increased risk of pain in the older adult population (OR = 1.53; 95% CI: 1.29–1.81; *p* < 0.001), even after adjusting for confounding variables [72]. Thus, HSG stands out as a useful and easy-to-measure clinical indicator, but also as a possible target for designing preventive interventions aimed at preserving functional performance in older adults with WP [73,74].

### 4.2. Practical Applications

This study’s results highlight the value of promoting strategies designed to preserve ADL performance and PA in older adults with WP. Early intervention on factors that threaten independence can encourage active aging. To achieve this, it is necessary to design well-structured evaluations and interventions, adapted to the characteristics and circumstances of each person, and adopt an integrated approach to the physical, cognitive, emotional, and social aspects that condition the daily performance of the population with WP. It should be considered a priority to set sustainable and accessible interventions tailored to this population, promoting MPA and muscle strengthening as strategies to promote active aging and relieve pressure on health and social systems.

Then, from a broad perspective, integrating such a vision and interventions into social and health systems should contribute to social and economic sustainability [5,10,18]. Promoting ADL performance maintenance in people with WP would help to reduce the need for care, optimize available resources, and reinforce health equity. This comprehensive person-centered approach, based on collaboration between disciplines (medicine, occupational therapy, physiotherapy, physical activity professionals, and others) [8], addresses the challenges of an aging population and should be aligned with the Sustainable Development Goals on health, inclusion, and social cohesion (SDGs 3, 10, and 11) [75].

### 4.3. Limitations and Future Lines

This study has several limitations. First, as it is a secondary cross-sectional study, (1) no cause–effect relationships between variables can be extracted but only relationships; and (2) the data were extracted from the Ninth Wave of the SHARE, so it was not specifically designed to examine the relationships between WP, ADLs, and physical condition, meaning that information relating to some of the variables analyzed may be missing. For example, in the case of pain, there was no information available on intensity or duration. Information on pain treatments (e.g., analgesics, physical therapy, occupational therapy, or psychological interventions) was not available, which may affect the results. The PA data were measured by self-report; no objective means were used, making them potentially biased. In addition, standard BMI values were used for categorization without specific adjustments for age. Data on lower limb strength, an important factor for mobility in older adults, were also not available. Although relative HGS is a validated and widely used indicator, it does not represent all the physical abilities involved in the performance of ADLs. The ADL information was collected by adapting two measures, the Katz Index (1963) for BADLs and the Lawton and Brody Scale (1969) for IADLs, which are quite old. Although they are widely used tools, the concept of ADLs has evolved over the years [12].

Different future lines are proposed. Firstly, it would be valuable to conduct longitudinal studies to analyze ADL functionality evolution in older people with WP, as well as the role played by PAF (MVA and VPA) and HGS in preventing ADLs and social participation decline; thus, this type of design would allow us to establish causal relationships. It would also be useful to incorporate more detailed data about pain, including its intensity, duration, specific location, and coping strategies, as these factors could influence the observed outcomes. In addition, the physical condition assessment could be expanded beyond HGS to include aspects such as lower limb strength, balance, and aerobic capacity, which are fundamental for daily functioning. It would also be relevant to explore the barriers and facilitators that older people with pain encounter in staying active, especially from a gender and social perspective (urban or rural, living at home or institutionalized, etc.).

## 5. Conclusions

A significant association was observed between MPA frequency and limitations in both BADL and IADL presence. People who performed MPA rarely or never had higher limitation prevalence in almost all the ADLs compared to those who performed it ‘very frequently’. In contrast, VPA showed no significant associations. Additionally, greater relative HGS was consistently associated with a lower risk of functional limitations, highlighting the protective role of muscle strength in maintaining ADL independence.

These findings are particularly relevant in the current context of population aging, with health and social systems under pressure, as they suggest that interventions focused on MPA and maintaining muscle strength can effectively contribute to delaying functional dependence. This improves the quality of life of older people with pain but also must be a key strategy for the sustainability of health and social systems by reducing the demand for care and the costs associated with loss of autonomy.

These findings are particularly relevant in the current context of global population aging, where health and social care systems face increasing strain. They suggest that interventions aimed at promoting MFA and preserving muscle strength may be effective in delaying the onset of functional dependence. Such approaches not only enhance the quality of life of older adults living with pain but also represent a key strategy for the sustainability of care systems by helping to reduce care demands and the economic burden associated with functional dependence.

## Figures and Tables

**Table 1:** Descriptive analysis of variables.

	Total n (1154)		Female n (819) 71%		Male n (335) 29%				
	Mdn	IQR	Mdn	IQR	Mdn	IQR	UM	df	*p*-Value
**Age (years)**	68	12	68	12	70	12	124,866		0.016 *
**Perception of loneliness**	4	3	4	3	4	3	103,751		0.139
**Hand Grip Ratio (** **kg/Weight)**	0.35	0.17	0.33	0.15	0.44	0.18	41,414		<0.001 ***
**Cognitive Functioning**	17	7	17	7	16	7	91,449		<0.001 ***
	**n**	**(%)**	**n**	**(%)**	**n**	**(%)**			
**Body Mass Index**									
Underweight	33	3%	22	3%	11	4%	2.14	3	0.544
Normal	333	31%	145	32%	88	28%			
Overweight	410	28%	283	37%	127	40%			
Obese	306	28%	217	28%	89	28%			
**Depression**									
No	40	40%	283	37%	126	46%	6.60	1	0.012
Yes	627	61%	479	63%	148	54%			
**Long-term illness**									
No	49	13%	110	13%	39	12%	0.63	1	0.426
Yes	1002	87%	708	87%	294	88%			
**Education level**									
None	41	4%	32	4%	9	3%	4.39	7	0.613
Pre-primary	215	1%	149	18%	66	20%			
Primary	230	20%	168	21%	62	19%			
Lower Secondary	447	29%	310	38%	137	41%			
Upper secondary	41	4%	28	3%	13	4%			
Post-secondary non-tertiary	169	15%	124	15%	45	13%			
First stage of tertiary education	7	1%	5	1%	2	1%			
Still in school	1	1%	0	0%	1	0%			
**Moderate Physical Activity**									
More than once a week	568	49%	410	50%	158	47%	10.65	3	0.014
Once a week	147	13%	114	14%	33	10%			
One to three times a month	91	8%	69	8%	22	7%			
Hardly ever, or never	345	30%	224	27%	121	36%			
**Vigorous Physical Activity**									
More than once a week	242	21%	175	21%	67	20%	3.64	3	0.303
Once a week	126	11%	97	12%	29	9%			
One to three times a month	86	8%	63	8%	23	7%			
Hardly ever, or never	696	61%	482	59%	214	64%			

n: number; Mdn: median; IQR: interquartile; UM: Mann–Whitney’s U; df: degree of freedom; * (*p* < 0.05); *** (*p* < 0.001).

**Table 2:** Instrumental Activities of Daily Living limitation prevalence by Moderate and Vigorous Physical Activity Frequency. Percentage of participants within each PAF category (out of 100% in that category).

Variable	Moderate Physical Activity Frequency											
	More Than Once a Week	Once a Week		1–3 Times a Month			Hardly Ever, or Never		*χ* ^2^	df	*p*	V
	n	(%)	n	(%)	n	(%)	n	(%)				
Meal preparation (hot meal)	18a	3%	4a	3%	4a	4%	132b	38%	250.768	3	<0.001 ***	0.467
Ability to use the phone	3a	1%	2a	1%	1a	1%	66b	19%	139.518	3	<0.001 ***	0.349
Responsibility for medication	8a	1%	3	2%	1	1%	87	25%	173.234	3	<0.001 ***	0.388
Housekeeping (house, garden)	107a	19%	42a	29%	22a	24%	207b	60%	170.768	3	<0.001 ***	0.386
Financial management	21a	4%	10a	7%	7a	8%	117b	34%	178.715	3	<0.001 ***	0.395
Community mobility	33a	6%	16a,b	11%	16b	18%	181c	53%	291.246	3	<0.001 ***	0.504
Doing laundry	15a	3%	5a	3%	6a	7%	134b	39%	257.323	3	<0.001 ***	0.473
Shopping	42a	7%	17a	12%	11a	12%	164b	48%	227.493	3	<0.001 ***	0.445
**Variable**	**Vigorous Physical Activity Frequency**											
	**More Than Once a Week**	**Once a Week**		**1–3 Times a Month**			**Hardly Ever, or Never**		***χ*** **^2^**	**df**	***p***	**V**
	**n**	**(%)**	**n**	**(%)**	**n**	**(%)**	**n**	**(%)**				
Meal preparation (hot meal)	5a	2%	1a	1%	0a	0%	152b	22%	97.581	3	<0.001 ***	0.292
Ability to use the phone	3a	1%	1	1%	0a	0%	68b	10%	32.036	3	<0.001 ***	0.18
Responsibility for medication	3a	1%	0a	0%	1a	1%	95b	14%	56.932	3	<0.001 ***	0.223
Housekeeping (house, garden)	41a	17%	19a	15%	14a	17%	304b	44%	92.994	3	<0.001 ***	0.285
Financial management	6a	3%	3a	2%	4a	5%	142b	20%	72.523	3	<0.001 ***	0.251
Community mobility	11a	5%	3a	2%	7a	8%	225b	32%	126.022	3	<0.001 ***	0.331
Doing laundry	5a	2%	1a	1%	2a	2%	152b	22%	92.329	3	<0.001 ***	0.284
Shopping	15a	6%	5a	4%	7a	8%	207b	29%	96.208	3	<0.001 ***	0.29

n: number; *χ*^2^: Chi-square; df: degree of freedom; *p*: *p*-value; *** (*p* < 0.001); V: Cramer’s V; a, b, c indicate post hoc differences in proportions.

**Table 3:** Odds ratios for reporting limitations in Basic Activities of Daily Living for Physical Condition variables (MPA, VPA, and HGS ratio) adjusted for sex, age, BMI, educational level, perception of loneliness, depression, and cognitive function.

Basic Activities of Daily Living Limitations					
		OR	(95% CI)		*p*
Dressing	Never vs. Very Frequently MPA	2.55	1.44	4.50	0.001 **
	Never vs. Very Frequently VPA	0.78	0.45	1.35	0.372
	Relative Handgrip Strength	0.03	0.00	0.22	<0.001 ***
Home Mobility	Never vs. Very Frequently MPA	9.08	2.99	27.55	<0.001 ***
	Never vs. Very Frequently VPA	1.47	0.38	5.65	0.579
	Relative Handgrip Strength	0.01	0.00	0.22	0.006 **
Bathing	Never vs. Very Frequently MPA	4.52	2.34	8.73	<0.001 ***
	Never vs. Very Frequently VPA	0.88	0.43	1.80	0.728
	Relative Handgrip Strength	0.01	0.00	0.11	<0.001 *
Feeding	Never vs. Very Frequently MPA	5.00	1.61	15.49	0.005 **
	Never vs. Very Frequently VPA	0.66	0.22	2.02	0.471
	Relative Handgrip Strength	0.00	0.00	0.00	<0.001 ***
Transferring	Never vs. Very Frequently MPA	2.28	1.13	4.62	0.022 *
	Never vs. Very Frequently VPA	0.87	0.44	1.75	0.703
	Relative Handgrip Strength	0.03	0.00	0.40	0.008 **
Toileting	Never vs. Very Frequently MPA	2.69	1.04	6.97	0.042 *
	Never vs. Very Frequently VPA	1.15	0.41	3.22	0.785
	Relative Handgrip Strength	0.00	0.00	0.10	<0.001 ***

OR: odds ratio; CI: confidence interval; *p* (*p*-value); * (*p* < 0.05); ** (*p* < 0.01); *** (*p* < 0.001). MPA: Moderate Physical Activity Frequency; VPA: Vigorous Physical Activity Frequency. Very frequently (more than once a week) was taken as reference.

**Table 4:** Odds ratios for reporting limitations in Instrumental Activities of Daily Living for Physical Condition variables (MPA, VPA, and handgrip strength ratio) adjusted for sex, age, BMI, educational level, perception of loneliness, depression, and cognitive function.

Instrumental Activities of Daily Living Limitations					
		OR	(95% CI)		*p*
Preparing meal	Never vs. Very Frequently MPA	4.05	1.57	10.48	0.004 **
	Never vs. Very Frequently VPA	2.67	0.73	9.80	0.139
	Relative Handgrip Strength	0.02	0.00	0.63	0.027 *
Shopping	Never vs. Very Frequently MPA	3.88	2.03	7.40	<0.001 ***
	Never vs. Very Frequently VPA	1.61	0.77	3.38	0.207
	Relative Handgrip Strength	0.02	0.00	0.20	0.001 **
Ability using the telephone	Never vs. Very Frequently MPA	12.10	1.76	83.29	0.011 *
	Never vs. Very Frequently VPA	0.35	0.05	2.55	0.298
	Relative Handgrip Strength	0.02	0.00	1.02	0.051
Housekeeping	Never vs. Very Frequently MPA	2.97	1.82	4.86	<0.001 ***
	Never vs. Very Frequently VPA	1.60	0.99	2.57	0.055
	Relative Handgrip Strength	0.02	0.00	0.11	<0.001 ***
Financial management	Never vs. Very Frequently MPA	3.14	1.21	8.18	0.019 *
	Never vs. Very Frequently VPA	0.99	0.32	3.11	0.990
	Relative Handgrip Strength	0.02	0.00	0.80	0.038 *
Responsibility for medication	Never vs. Very Frequently MPA	9.20	2.20	38.47	0.002 **
	Never vs. Very Frequently VPA	1.33	0.24	7.34	0.744
	Relative Handgrip Strength	0.03	0.00	2.94	0.129
Community mobility	Never vs. Very Frequently MPA	4.97	2.62	9.42	<0.001 ***
	Never vs. Very Frequently VPA	1.98	0.89	4.40	0.094
	Relative Handgrip Strength	0.01	0.00	0.17	<0.001 ***
Doing laundry	Never vs. Very Frequently MPA	7.53	2.97	19.06	<0.001 ***
	Never vs. Very Frequently VPA	1.61	0.50	5.25	0.426
	Relative Handgrip Strength	0.00	0.00	0.10	<0.001 ***

OR: odds ratio; CI: confidence interval; *p* (*p*-value); * (*p* < 0.05); ** (*p* < 0.01); *** (*p* < 0.001). MPA: Moderate Physical Activity Frequency; VPA: Vigorous Physical Activity Frequency. Very frequently (more than once a week) was taken as reference.

## Data Availability

Data are available upon request through the SHARE Data Platform website (www.share-eric.eu/ accessed on 1 March 2020).

## Abbreviations

The following abbreviations are used in this manuscript:

WP	Widespread Pain
ADLs	Activities of Daily Living
BADLs	Basic Activities of Daily Living
IADLs	Instrumental Activities of Daily Living
SHARE	The Survey of Health, Ageing and Retirement in Europe
PA	Physical Activity
HGS	Handgrip Strength
MPA	Moderate Physical Activity
VPA	Vigorous Physical Activity
